# Transcriptome Analysis of *Culex pipiens quinquefasciatus* Larvae Exposed to a Semi-Lethal Dose of Vermistatin

**DOI:** 10.3390/tropicalmed10020031

**Published:** 2025-01-22

**Authors:** Junhui Chen, Zhiyong Xu, Feiying Yang, Jian Yang, Wendong Kuang, Jianghuai Li, Yaqi Wang, Liang Jin

**Affiliations:** 1 Key Laboratory of Modern Preparation of TCM, Jiangxi University of Chinese Medicine, Ministry of Education, Nanchang 330006, China; allenchen0426@gmail.com; 2 Institute of Microbiology, Jiangxi Academy of Sciences, Nanchang 330022, China; jemappelleyangjian@zju.edu.cn (J.Y.); kuangwendong@163.com (W.K.); jeremy_leakey@sina.com (J.L.); 3 Institute of Applied Chemistry, Jiangxi Academy of Sciences, Nanchang 330022, China; zhiyongxuconfident@hotmail.com; 4 Institute of Biological Resources, Jiangxi Academy of Sciences, Nanchang 330022, China; jxaasyfy@163.com; 5 Jiangxi Provincial Key Laboratory of Plantation and High Valued Utilization of Specialty Fruit Tree and Tea, Institute of Biological Resources, Jiangxi Academy of Sciences, Nanchang 330022, China

**Keywords:** mosquito, vermistatin, RNA-seq, vector control, gene expression, detoxification gene

## Abstract

*Culex pipiens quinquefasciatus* is a notorious vector transmitting severe diseases such as Zika virus and West Nile virus to humans worldwide. Vermistatin is a type of funicon-like compound and was first isolated from *Penicillin vermiculatum* in the 1970s. Vermistatin has shown promising activity against *Cx. p. quinquefasciatus* larvae in our previous research. Here, we conducted a transcriptomic analysis of *Cx. p. quinquefasciatus* larvae treated with a median lethal concentration of 28.13 mg/L vermistatin. Differential expression analysis identified 1055 vermistatin-responsive genes, with 477 downregulated and 578 upregulated. Gene Ontology annotation and enrichment analysis revealed the metabolic process to be the most significantly affected biological process, the membrane to be the most significantly affected cellular component, and catalytic activity to be the most significantly affected molecular function. Kyoto Encyclopedia of Genes and Genomes pathway analysis classified the differential expression genes into six major categories, with metabolism and organismal systems being the most enriched. Fifty-five pathways were significantly enriched, with the hematopoietic cell lineage, renin–angiotensin system, cholesterol metabolism, and peroxisome proliferator-activated receptor signaling pathways among the top altered pathways. Furthermore, 32 potential detoxification-related genes were differentially expressed, with 3 cytochrome P450s, 2 ABC transporters, and 1 UGT induced by vermistatin. This study provides insights into the molecular mechanisms of vermistatin’s action against *Cx. p. quinquefasciatus* larvae, highlighting potential targets for novel mosquito control strategies.

## 1. Introduction

Mosquitoes are notorious pest vectors transmitting various diseases and causing numerous deaths worldwide. *Culex pipiens quinquefasciatus* is mainly distributed in the urban and rural areas of Southern China [1]. As an opportunistic blood feeder, *Cx. p. quinquefasciatus* commonly feeds on humans in urban areas. The wide distribution of *Cx. p. quinquefasciatus* poses to the threat of the introduction and transmission of various diseases such as Japanese encephalitis virus (JEV) and Zika virus (ZIKV) [2,3].

Vector control has been globally applied to reduce the spread of diseases due to the lack of effective vaccines [4]. Pesticides have been proven to be the primary approach for vector control. Insecticide Resistance Action Committee (IRAC) summarizes a series of modes-of-action classifications of insecticides against mosquitoes (https://irac-online.org/mode-of-action/ assessed on 1 December 2024). Nerve and muscle, growth and development, the midgut, and respiration are major targets for mosquitoes. Preidgeon et al. [5] tested 19 pesticides against three mosquito species with different modes of action and found that *Cx. p. quinquefasciatus* adults were susceptible to DNOC, azocyclotin, chlorfenapyr, carbaryl, spinosad, imidaclorid, diazinon, abamectin, and permethrin. Maintaining the effectiveness of pesticides is usually accompanied by abuse and eventually increases resistance. Insecticide resistance decreased the efficacy of the major class of pyrethroids, which poses the threat of a rising incidence of malaria and fatalities [6].

Vermistatin, a type of funicon-like compound, was first isolated from *Penicillin vermiculatum* and its structure elucidated in 1979 [7,8]. In addition to *Penicillin*, vermistatin has been isolated from other genera, such as *Aspergillus* [9], *Cladosporium* [10], *Guignardia* [11], *Phoma* [12], and *Talaromyces* [13,14]. The functions of vermistatin have been gradually discovered. For example, vermistatin has proven its antiviral activity against canine coronaviruses (CCoV) in A72 cells [15], its enzyme inhibitory effect on α-glucosidase [16,17], and its growth inhibition activities against first-instar larvae of *Helicoverpa armigera* [10]. Moreover, vermistatin could be used as a synergist reinforcing the inhibition activity of miconazole against *Candida albicans* [13].

In our previous study, we successfully isolated vermistatin from ethyl acetate crude extract from *Talaromyces* sp. and proved its toxicity against *Cx. p. quinquefasciatus* larvae [14]. The lethal concentration of 50% (LC_50_) of vermistatin against *Cx. p. quinquefasciatus* was 28.13 mg/L, which was higher other commercial products such as propoxur (0.29 mg/L) [18] and spinosad (0.671 mg/L) [19]. Hence, it would be meaningful for further uses of vermistatin and/or its derivates to reveal the molecular mechanism of vermistatin against *Cx. p. quinquefasciatus*. In this study, we conducted a transcriptomic analysis to uncover the comprehensive alterations in gene expression in *Cx. p. quinquefasciatus* subjected to 28.13 mg/L of vermistatin, aiding in elucidating the molecular basis of vermistatin’s action against *Cx. p. quinquefasciatus*.

## 2. Materials and Methods

### 2.1. Mosquitoes and Vermistatin

*Cx. p. quinquefasciatus* was reared in the insect-rearing room at Nanchang Center for Disease Control and Prevention, Jiangxi Province (China). Mosquitoes were originally collected inn Nanchang and subsequently reared in the laboratory without exposure to pesticides. Larvae were fed with a mixture of yeast and lactose albumin in equal weight in an insect-rearing room with 26 ± 1 °C, 65 ± 10% relative humidity (RH), and a photoperiod of 12 h light and 12 h dark. Mosquito adults were provided with a damp mass of cotton with sugar solution (10%) and sheep blood from Mozzie Blood Feeder (Jiangmen Junhan Technology Co., Ltd., Jiangmen, China). Vermistatin (≥98.0%) was purchased from Sichuan ChemConst Biotechnology Co., Ltd. (Chengdu, China).

### 2.2. Larvae Treatment

Bioassays of fourth-instar larvae were conducted based on the requirements of the WHO (2005). Previous results showed that the LC_50_ of vermistatin to *Cx. p. quinquefasciatus* larvae was 28.13 mg/L [14]. First, vermistatin (5 mg) was dissolved into dimethyl sulfoxide (DMSO) (1.78 mL) to make a vermistatin stock solution (2.813 mg/mL). Then, the vermistatin stock solution (500 μL) was added to distilled water (50 mL) to make a final vermistatin concentration (28.13 mg/L), while the control group was treated with DMSO (500 μL). Forty fourth-instar larvae were chosen as a replicate of the treatment group and control group; each treatment and control group contained 3 biological replicates. After 24 h, 20 larvae were alive in the treatment groups and 40 larvae were alive in the control groups. Ten living larvae were collected, immediately frozen in liquid nitrogen, and then stored in a −80 °C refrigerator for further RNA extraction in all groups.

### 2.3. Transcriptomics Sequencing

Based on the instructions of the TRIzol^®^ Reagent (Invitrogen, Carlsbad, CA, USA), total RNA from the fourth-instar larvae of *Cx. p. quinquefasciatus* was extracted. Sampling was performed at Shanghai Majorbio Bio-pharm Biotechnology Co., Ltd. (Shanghai, China), for RNA purification, reverse transcription, library construction, and sequencing. RNA concentration and purity were assessed using a NanoDrop 2000 (Thermo Fisher Scientific, Sunnyvale, CA, USA), RNA integrity was assessed through agarose gel electrophoresis, and the RNA quality number (RQN) value was determined using an Agilent 5300 bioanalyzer (Agilent Technologies, Santa Clara, CA, USA). To create transcriptome libraries, 1 μg of total RNA (RQN value >6.5 and OD_260/280_ ratio between 1.8 and 2.2) by Illumina^®^ Stranded mRNA Prep, Ligation (San Diego, CA, USA) was utilized. Qubit 4.0 (Thermo Fisher Scientific, Sunnyvale, CA, USA) was used for preliminary quantification, a NovaSeq X Plus platform (PE150) (Illumina, San Diego, CA, USA)was used for the sequencing library, and FASTQ (version 0.19.6) was used to trim and carry out quality control for the raw paired-end reads [20]. The clean reads with high quality were mapped to the *Cx. p. quinquefasciatus* genome (https://www.ncbi.nlm.nih.gov/datasets/genome/GCF_000209185.1/ accessed on 1 December 2024) with orientation mode using HISAT2 (version 2.2.1) [21], and assembled using StringTie (version 2.2.2) [22].

### 2.4. Differentially Expressed Gene RNA-Seq Analysis

Differentially expressed genes (DEGs) were identified by DESeq2 (version 3.19) [23]. DEGs with |log_2_FC| ≧ 1, FDR < 0.05, and FPKM of vermistatin or Calvin Klein (CK) treatment >10 (DEGseq) were considered to be significant. Moreover, functional annotation and enrichment analysis, namely Gene Ontology (GO) and Kyoto Encyclopedia of Genes and Genomes (KEGG), were performed using Goatools software (version 1.4.4) and the Python SciPy package (version 1.4.1), respectively. Significant enrichment in GO terms and metabolic pathways was identified based on a Bonferroni-corrected *p*-value < 0.05 in comparison with the whole-transcriptome background.

### 2.5. Reverse Transcription Quantitative PCR

Twelve DEGs were selected for qPCR analysis to confirm the differential expression observed in the RNA-Seq. The RNAs of fourth-instar larvae from both the vermistatin treatment and CK were extracted using Invitrogen TRIzol^®^ reagent and quantified using a Nanodrop 2000. A HiScript II Q RT SuperMix for qPCR kit (Vazyme, Nanjing, China) was used to synthesize cDNA. *RPL8* of *Cx. p. quinquefasciatus* was used as an internal reference [24]. Primers of DEGs were designed using DNAMAN software (version 8.0), and the primer sequences are listed in Table 1. A 20 μL PCR reaction system was made using 10 μL 2 × ChamQ Universal SYBR qPCR Master Mix (Vazyme, Nanjing, China), 7.2 μL ddH_2_O, 2 μL cDNA template, and 0.4 μL (10 MM) of each primer. A standard qRT-PCR program was performed in the Applied Biosystems QuantStudio 7 Flex Real-Time PCR system (Thermo Fisher Scientific, Sunnyvale, CA, USA). The 2^−∆∆Ct^ method was used to determine the relative gene expression levels [25]. TBtools software (version 1.1043, China) was used to draw a heatmap regarding the patterns of differential gene expressions among different treatments [26].

## 3. Results

### 3.1. Analysis of Transcriptome Sequencing

The transcriptomes of *Cx. p. quinquefasciatus* larvae treated with vermistatin (Ver1, Ver 2, and Ver 3) and the control (CK1, CK2, and CK3) were sequenced and deposited as BioProject SAMN43359958-SAMN 43359963 in the NCBI SRA database. Total clean reads ranging from 48,129,304 to 53,963,162 were ultimately obtained when adaptor sequences, ambiguous “N” nucleotides, and low-quality sequences were removed. The GC counts were approximately 50%, ranging from 50.26 to 50.82%; the Q20 ratios were >98%; and the Q30 ratios were >96% (Table 2).

### 3.2. Differentially Expressed Genes (DEGs)

**Figure 1:**
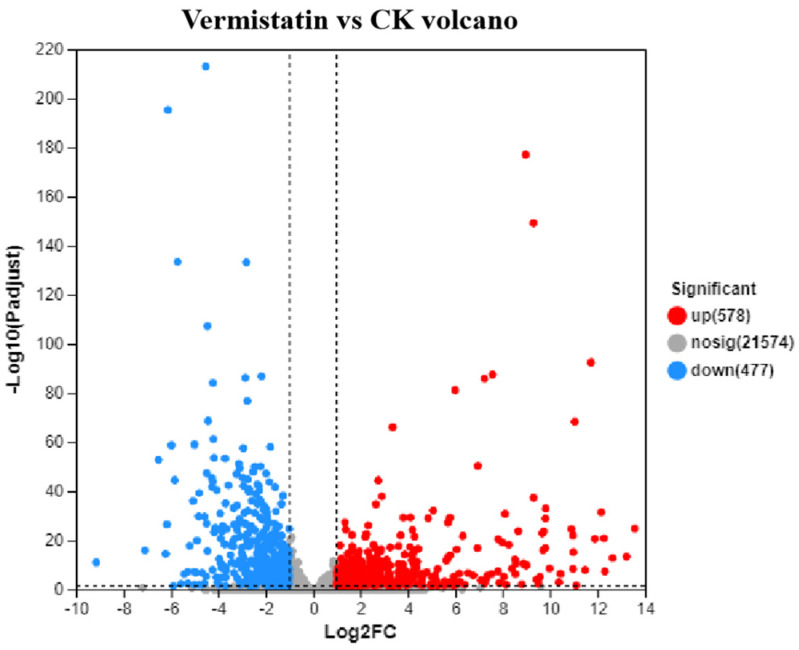
Differentially expressed genes (DEGs) in the *Culex pipiens quinquefasciatus* larvae between vermistatin- and CK-treated samples. Dots indicate individual genes: red dots represent significantly upregulated genes, blue dots significantly downregulated genes, and gray dots non-significant differentially expressed genes.

In total, 1055 vermistatin-responsive DEGs were found in the *Cx. p. quinquefasciatus* transcriptomes treated with vermistatin. Among them, 477 genes were downregulated and 578 genes were upregulated (Figure 1).

### 3.3. GO Annotation and Enrichment Analysis

**Figure 2:**
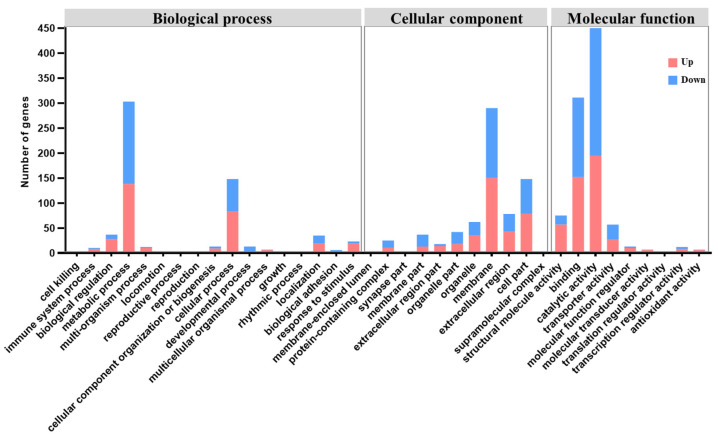
Gene Ontology cluster diagram of differentially expressed genes.

The two biological process GO terms with the highest number of DEGs were the metabolic process, with 138 upregulated and 165 downregulated DEGs, and the cellular process, with 83 upregulated and 65 downregulated DEGs. Membrane, with 150 upregulated and 140 downregulated DEGs; cell part, with 79 upregulated and 69 downregulated DEGs; and extracellular region, with 43 upregulated and 35 downregulated DEGs, were the top three cellular component GO terms. The top two regulated DEGs of molecular function were catalytic activity (195 upregulated and 255 downregulated DEGs) and binding (152 upregulated and 159 downregulated DEGs) (Figure 2).

**Figure 3:**
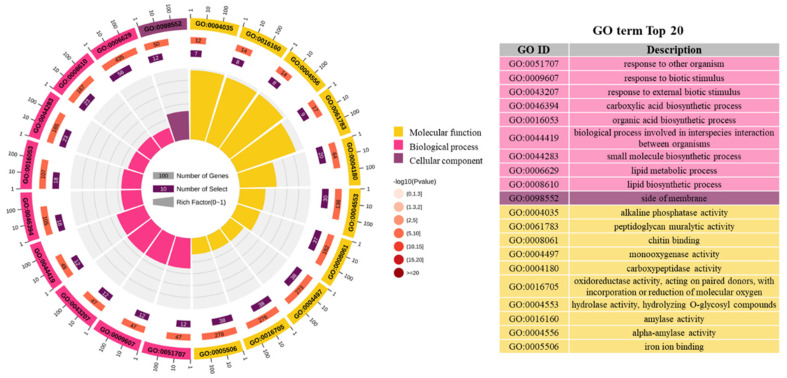
A Gene Ontology (GO) term enrichment analysis of the selected genes (top 20). From outside to inside, the first lap indicates the classification of enrichment, and the outside circle is a coordinate ruler of the number of genes. The second lap indicates the number of genes and *p*-values. The third lap indicates a bar chart of the selected genes. The fourth lap indicates the richness factor of each GO term. Different colors represent different classifications.

Ten of the top twenty regulated DEGs were molecular function, followed by the biological process, with nine regulated DEGs, and only one regulated DEG was cellular function (Figure 3). The DEGs were involved in 10 molecular function GO terms (GO:0004035, GO:0061793, GO0008061, GO:0004497, GO:0004180, GO:0016705, GO:0004553, GO:0016160, GO:0004556, and GO:0005506), 9 biological process GO terms (GO:0051707, GO:0009607, GO:0043207, GO:0046394, GO:0016053, GO:0044419, GO:0044283, GO:0006629, and GO:0008610), and 1 cellular component GO term (GO:0098552).

### 3.4. KEGG Annotation and Enrichment Analysis

**Figure 4:**
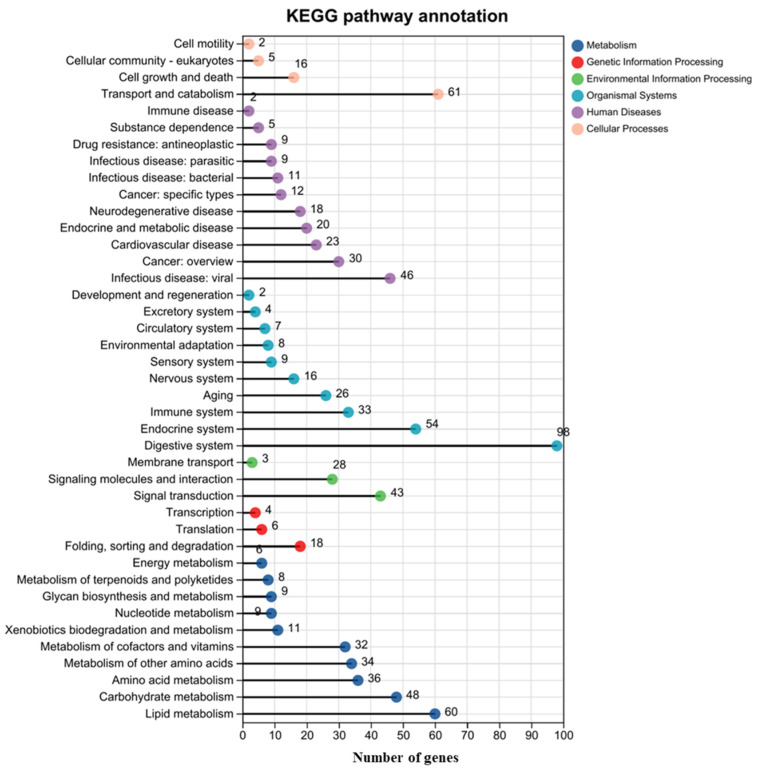
Annotation of Kyoto Encyclopedia of Genes and Genomes (KEGG). The ordinate indicates the KEGG pathways, and the abscissa is the number of differential genes annotated to the KEGG pathway.

Six classes, metabolism, genetic information processing, environmental information processing, organismal systems, human diseases, and cellular processes, were classified by the KEGG pathway annotation (Figure 4). Most of the DEGs were involved in metabolism and organismal systems. Among them, the digestive system and endocrine system were the most significantly changed sub-classes under organismal systems, while lipid metabolism and carbohydrate metabolism were the most significantly changed sub-classes under the metabolism class. Transport and catabolism was the most significantly enriched sub-class under cellular processes, and signal transduction was the most significantly enriched in environmental information processing.

**Figure 5:**
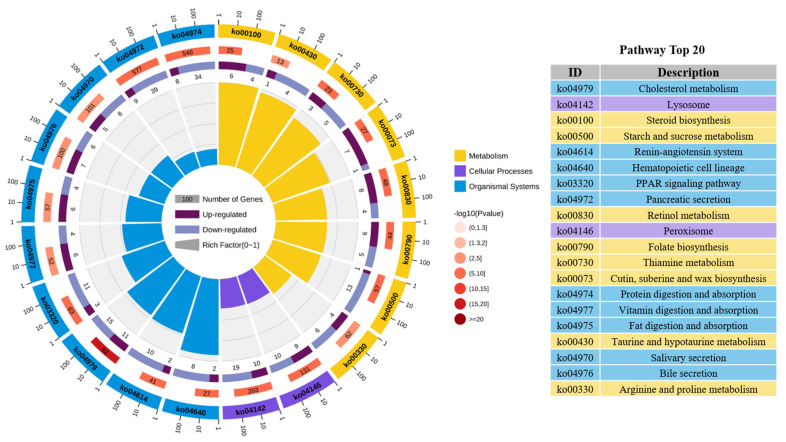
Kyoto Encyclopedia of Genes and Genomes pathway enrichment analysis of differentially expressed genes (top 20). From outside to inside, the first lap indicates the classification of enrichment, and the outside circle is a coordinate ruler of the number of genes. The second lap indicates the number of genes and *p*-values. The third lap contains a bar chart of the proportion of differential genes that have been adjusted up and down; dark purple represents the proportion of upregulated genes, while light purple represents the proportion of downregulated genes. The fourth lap indicates the richness factor of each pathway. Different colors represent different classifications.

Fifty-five different pathways of *Cx. p. quinquefasciatus* were significantly enriched between the vermistatin treatment and CK (*p*-value < 0.05). The top 20 pathways belonged to three classes (Figure 5). Ten pathways (hematopoietic cell lineage, renin–angiotensin system, cholesterol metabolism, peroxisome proliferator-activated receptor (PPAR) signaling pathway, vitamin digestion and absorption, fat digestion and absorption, bile secretion, salivary secretion, pancreatic secretion, protein digestion and absorption) were under organismal systems, eight pathways (steroid biosynthesis, taurine and hypotaurine metabolism, thiamine metabolism, cutin, suberine and wax biosynthesis, retinol metabolism, folate biosynthesis, starch and sucrose metabolism, arginine and proline metabolism) were under metabolism, and two pathways (lysosome, peroxisome) were under cellular processes.

### 3.5. RNA-Sequencing Validation

**Figure 6:**
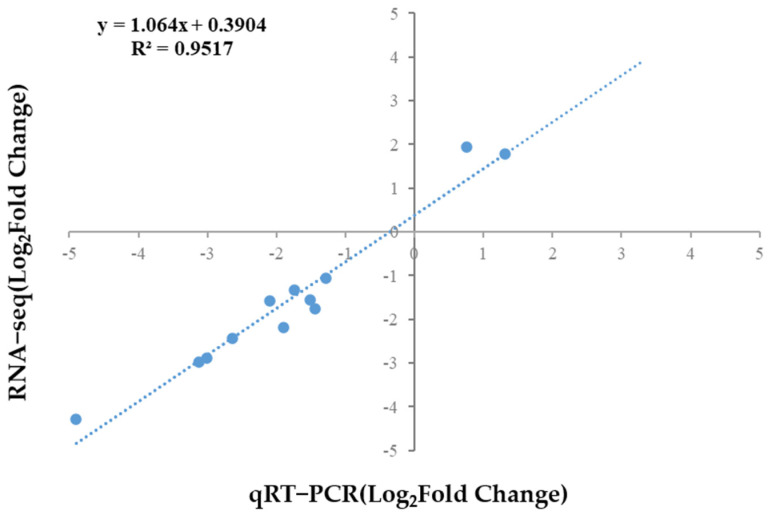
Analysis of RNA-seq results versus qRT-PCR: a comparison based on Log2 (Fold Change) data from 12 randomly selected genes.

Twelve DEGs were randomly chosen, and the R^2^ (0.9517) of the qRT-qPCR results confirmed the reliability of the RNA-seq results (Figure 6).

### 3.6. Potential Detoxification and α-Glucosidase Genes

**Figure 7:**
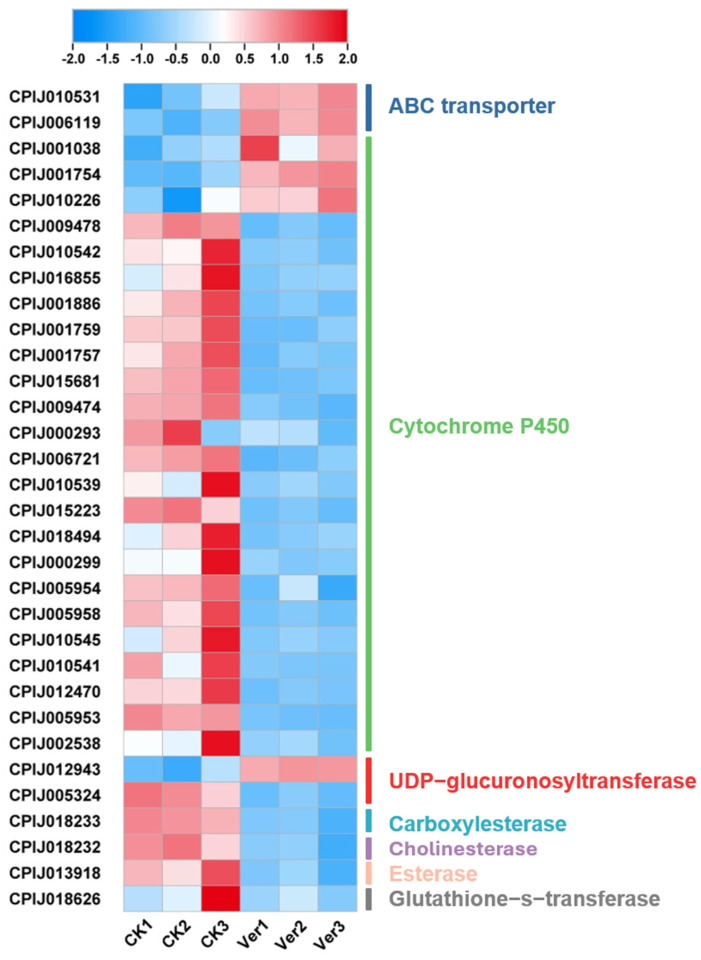
Potential detoxification gene expression of *Culex pipiens quinquefasciatus* treatment with vermistatin.

A total of 32 potential detoxification genes, including 2 ATP-binding cassette transporters (ABC transporters), 24 cytochrome P450s, 2 UDP-glucuronosyltransferases (UGTs), 1 carboxylesterase (CarE), 1 cholinesterase (ChE), 1 esterase (EST), and 1 glutathione-s-transferase (GST), were differentially expressed between vermistatin-treated and CK-treated samples of *Cx. p. quinquefasciatus* larvae (Figure 7). Among them, six genes, including two ABC transporters (*CPIJ010531* and *CPIJ006119*), three cytochrome P450s (*CPIJ001038*, *CPIJ001754*, and *CPIJ010226*), and one UGT (*CPIJ012943*), were induced by vermistatin, and the other 26 genes were inhibited.

Four α-glucosidase genes (*CPIJ008904*, *CPIJ012204*, *CPIJ013171*, and *CPIJ013172*) were inhibited by vermistatin.

## 4. Discussion

Vermistatin has been isolated from various fungal metabolites [9,11,13] and has many functions, including as a potential insecticide. Vermistatin, isolated from *Cladosporium* sp., showed growth inhibition activities against first-instar larvae of *Helicoverpa armigera*, with an IC_50_ of 150 μg/mL [10]. In our previous study, vermistatin was isolated from *Talaromyces* sp. and showed toxic activity against *Cx. p. quinquefasciatus*, with an LC_50_ of 28.13 mg/L [14], but the molecular mechanism of vermistatin against *Cx. p. quinquefasciatus* remains unclear.

Here, comparative transcriptome data from *Cx. p. quinquefasciatus* larvae were acquired, and the comprehensive response to vermistatin with semi-lethal levels was determined. A total of 1055 DEGs were identified with 477 down- and 578 upregulated genes, revealing a homeostatic response to insecticides through a balance of these genes’ upregulation or downregulation [27]. The enriched GO terms obtained for the DEGs occurred primarily among the molecular functions and biological process, where they represented three functional categories, catalytic activity, binding, and metabolic process, suggesting that vermistatin may directly interact with enzymes and/or proteins, modulate their activity, and ultimately alter the physiology of mosquitoes. KEGG annotation showed that DEGs enriched the steroid biosynthesis, taurine and hypotaurine metabolism, thiamine metabolism, and hematopoietic cell lineage pathways, indicating that vermistatin may disrupt the biosynthesis and metabolic pathways and ultimately cause the death of mosquito larvae. In addition, DEGs enriched cutin, suberine, and wax biosynthesis in our study, meaning that the proteins in epidermal pathways in the defense mechanism of *Cx. p. quinquefasciatus* were activated by vermistatin. Other studies also showed that cuticular protein thickening may protect mosquitoes from insecticides by leading to a reduction in insecticide penetration [28,29,30].

Insects have developed numerous complex detoxification mechanisms to survive exposure to various poisonous xenobiotics/pesticides [31,32]. Detoxification enzymes, such as cytochrome P450s, ABC, and UGTs, play an important role in insecticide detoxification and resistance development. Xenobiotics/insecticides can disrupt the enzymatic equilibrium for basic physiological activities [33,34]. Numerous detoxification-related pathways, such as cytochrome P450 enzymes, starch, and sucrose metabolism, and retinol metabolism, are deployed to deal with insecticide toxicities in insects [35].

In this study, three cytochrome P450 unigenes, *CYP12A2* (*CPIJ010226*), *CYP4V3* (*CPIJ001754*), and *CYP18A1* (*CPIJ001038*), were upregulated by vermistatin (3.8-, 2.9-, and 2.6-fold, respectively). The higher cytochrome P450 levels indicate that mosquitoes detoxify xenobiotics through metabolization to reduce damage [36], demonstrating the possibility of using *CYP12A2*, *CYP4V3*, and *CYP18A1* in xenobiotic detoxification. Cytochrome P450s in *Cx. p. quinquefasciatus* play an important role in the resistance of pyrethroid [37,38] and permethrin [36,39]. Five cytochrome P450 genes were upregulated >2.5-fold in pyrethroid-resistant *Cx. p. quinquefasciatus* compared to the susceptible strain [38]. The cytochrome P450s in the permethrin-resistant *Cx. p. quinquefasciatus* strain showed ~3-fold greater resistance than the permethrin-susceptible strain [36]. In addition, data from many other studies have indicated the important role of cytochrome P450s in insecticide detoxification in *Cx. p. pallens* [40], *Anopheles gambiae* [41], and *Aedes aegypti* [42].

In this study, one *UGT2C1* (*CPIJ012943*) was induced by vermistatin (3.5-fold). Overexpression of UGTs in insecticide resistance has also been found in other species, such as *Aedes* [43] and *Anopheles* [44,45], indicating that UGTs may be crucial across multiple vector species. ABC transporters, which function as ATP-dependent transporters of various substrates across biological membranes, have been identified in 70 genes in *Cx. p. quinquefasciatus* [46] and 63 genes in *Cx. p. pallens* [47]. Here, two ABC transporters (*CPIJ010531* and *CPIJ006119*) were induced by vermistatin (2.9- and 3.1-fold), suggesting that these genes encoding detoxification-related enzymes are candidates for vermistatin metabolism. So, further experimental evidence is needed to confirm whether these detoxification-related genes are involved in the detoxification of vermistatin.

Vermistatin can inhibit α-glucosidase activity [16,17], which is consistent with our results. Vermistatin significantly inhibited 4 α-glucosidase genes (*CPIJ008904*, *CPIJ012204*, *CPIJ013171*, *CPIJ013172*) in this study. Vermistatin may affect mosquito digestion by inhibiting the activity of digestive enzymes, eventually leading to the death of *Cx. p. quinquefasciatus*. Further research on the functions of these four α-glucosidase genes should be considered to reveal the mode of action of vermistatin against *Cx. p. quinquefasciatus*.

Future research should elucidate the specific mechanisms of vermistatin-mediated toxicity, such as its interaction with key enzymes and proteins, and identify the genetic determinants of vermistatin tolerance in mosquito populations. In addition, an investigation of vermistatin’s effects on other mosquito species and an exploration of vermistatin’s toxicity and environmental impact could be the focus of future studies. Moreover, we only focused on 24 h treatment with vermistatin, but we lacked information regarding the time course. Studying the changes in gene levels at different time points would lay a more stable foundation to identify the mode of action by providing a better understanding of specific genes responding to xenobiotics.

## 5. Conclusions

We assessed the role of vermistatin in *Cx. p. quinquefasciatus* by comparing transcriptomes and identified a total of 1055 differentially expressed genes (DEGs) in response to vermistatin. Of these, 477 genes were downregulated, while 578 were upregulated. Notably, these DEGs were predominantly enriched in metabolic and organismal system pathways. Further investigation into potential detoxification-related genes revealed that vermistatin induced the expression of three cytochrome P450 enzymes, two ABC transporters, and one UGT. These results are fundamental bases for future studies on insecticide resistance and the molecular mechanism of insecticide detoxification in *Cx. p. quinquefasciatus*.

## Figures and Tables

**Table 1:** Differentially expressed genes and their primer sequences used in qRT-PCR.

Gene Identity	Forward Primer Sequence (5′–3′)	Reverse Primer Sequence (5′–3′)
*RPL8*	GCTGGCCGAAGGTGCGTGGT	TTGCGACCTGGCGGCGTTCC
*CPIJ000293*	TGCTGCTGTGCTCCACTC	GTCTTCGCTGGTGCCACT
*CPIJ001759*	CCGCCGGAAGATGCTTGA	GACTCAGTGGCTTGCCGT
*CPIJ001886*	GCTCGCGGAACTGCATTG	GAGCGATTCCCCGGGAAG
*CPIJ005954*	TGACGATGGTGCGCAGTT	ATGCTACCCTCAGCCGGA
*CPIJ006721*	GCGGATGGTCGAGATGCA	TTGCCTTCGCCATCAGCA
*CPIJ010226*	TCGGATGTGCATCGGACG	GCGCTCAAGATGTGCAGC
*CPIJ010541*	AACGTGAGGCGCATGGAA	ACCGTTGCGAATCCTGCA
*CPIJ010545*	TTTCGGCGCCAGTGACAT	CAACGTCGATCGCATGCG
*CPIJ012943*	GATGGAGCGACGGATGGG	TCGCTACCAACGCTTGCA
*CPIJ013918*	TCAGCGCGCGATCGTAAT	CCCGTTCCATCCGAGAGC
*CPIJ018233*	TGTGGAGGCTCTGCGTTG	ACCCGGTTGCCTTGTGAG
*CPIJ018494*	GCGCTGGTGGTGTTTGTG	CCAGATTGCCCGTCAGCA

**Table 2:** Statistical data of the transcriptome for 6 mRNA libraries from the CK- and vermistatin-treated samples.

Samples	Raw Reads	Clean Reads	Clean Bases	GC (%)	Q20 (%)	Q30 (%)
CK1	52,305,134	51,926,458	7,774,431,980	50.78	98.83	96.31
CK1	54,364,436	53,963,162	8,088,174,870	50.82	98.81	96.22
CK1	50,451,260	50,074,842	7,509,214,644	50.63	98.82	96.27
Ver1	48,439,190	48,129,304	7,208,948,179	50.44	98.84	96.32
Ver2	51,960,662	51,575,346	7,723,510,476	50.26	98.76	96.09
Ver3	50,653,416	50,240,476	7,532,355,356	50.45	98.8	96.21

## Data Availability

The raw sequence data of RNA-seq have been deposited as BioProject SAMN43359958-SAMN 43359963 in the NCBI SRA database.
