# Correction: Chen et al. Transcriptome Analysis of *Culex pipiens quinquefasciatus* Larvae Exposed to a Semi-Lethal Dose of Vermistatin. *Trop. Med. Infect. Dis.* 2025, *10*, 31

**DOI:** 10.3390/tropicalmed10030059

**Published:** 2025-02-25

**Authors:** Junhui Chen, Zhiyong Xu, Feiying Yang, Jian Yang, Wendong Kuang, Jianghuai Li, Yaqi Wang, Liang Jin

**Affiliations:** 1 Key Laboratory of Modern Preparation of TCM, Jiangxi University of Chinese Medicine, Ministry of Education, Nanchang 330006, China; allenchen0426@gmail.com; 2 Institute of Microbiology, Jiangxi Academy of Sciences, Nanchang 330022, China; jemappelleyangjian@zju.edu.cn (J.Y.); kuangwendong@163.com (W.K.); jeremy_leakey@sina.com (J.L.); 3 Institute of Applied Chemistry, Jiangxi Academy of Sciences, Nanchang 330022, China; zhiyongxuconfident@hotmail.com; 4 Institute of Biological Resources, Jiangxi Academy of Sciences, Nanchang 330022, China; jxaasyfy@163.com; 5 Jiangxi Provincial Key Laboratory of Plantation and High Valued Utilization of Specialty Fruit Tree and Tea, Institute of Biological Resources, Jiangxi Academy of Sciences, Nanchang 330022, China

## Removal of Two Authors

In the original publication [1], Guodong Niu and Jun Li were listed as the seventh and eighth authors, but they require the removal of their names and the following affiliation: “Department of Biological Sciences, Florida International University, Miami, FL 33199, USA; gniu@fiu.edu (G.N.); lij@fiu.edu (J.L.)”. This has been agreed upon by all authors.

## Author Contributions Statement Correction

The updated author contributions are: “Conceptualization, Y.W.; Data Curation, W.K.; Formal Analysis, J.Y.; Funding Acquisition, J.C. and Z.X.; Investigation, J.C. and F.Y.; Methodology, Z.X. and J.Y.; Project Administration, Y.W.; Resources, L.J.; Software, F.Y.; Supervision, L.J.; Validation, J.L.; Visualization, J.L.; Writing—Original Draft, J.C. and Z.X.; Writing—Review and Editing, Y.W., and L.J. All authors have read and agreed to the published version of the manuscript”.

## Funding Correction

In the original publication, “the Ganpo talent support plan [Grant numbers 20232BCJ25027 and 20232BCJ25083]” were associated with Guodong Niu and Jun Li, so they should be removed.

The authors state that the scientific conclusions are unaffected. This correction was approved by the Academic Editor. The original publication has also been updated.
